# Long-Term Changes in Human Colonic *Bifidobacterium* Populations Induced by a 5-Day Oral Amoxicillin-Clavulanic Acid Treatment

**DOI:** 10.1371/journal.pone.0050257

**Published:** 2012-11-27

**Authors:** Irène Mangin, Christophe Lévêque, Fabien Magne, Antonia Suau, Philippe Pochart

**Affiliations:** 1 Laboratoire de Biologie, Conservatoire national des arts et métiers, Paris, France; 2 EA 4065, Faculté des Sciences Pharmaceutiques et Biologiques, Université Paris Descartes, Paris, France; 3 Laboratoire Adaptation et Pathogénie des Micro-organismes, Université Joseph Fourier, Grenoble, France; University of Malaya, Malaysia

## Abstract

The objective of this study was to assess the possible modifications due to amoxicillin-clavulanic acid (AMC) treatment on total bacteria and on *Bifidobacterium* species balance in human colonic microbiota. Eighteen healthy volunteers (19 to 36 years old) were given a 875/125 mg dose of AMC twice a day for 5 days. Fecal samples were obtained before and after antibiotic exposure. After total DNA extraction, total bacteria and bifidobacteria were specifically quantified using real-time PCR. Dominant species were monitored over time using bacterial and bifidobacterial Temporal Temperature Gradient gel Electrophoresis (TTGE). At the end of AMC exposure, total bacterial concentrations as well as bifidobacteria concentrations were significantly reduced compared to before AMC exposure:10.7±0.1 log_10_ 16S rRNA gene copies/g *vs* 11.1±0.1 log_10_ (p = 0.003) and 8.1±0.5 log_10_ 16S rRNA gene copies/g *vs* 9.4±0.3 log_10_ (p = 0.003), respectively. At the same time, the mean similarity percentages of TTGE bacteria and TTGE bifidobacteria profiles were significantly reduced compared to before AMC exposure: 51.6%±3.5% *vs* 81.4%±2.1% and 55.8%±7.6% vs 84.5%±4.1%, respectively. Occurrence of *B. adolescentis*, *B. bifidum* and *B. pseudocatenulatum/B. catenulatum* species significantly decreased. Occurrence of *B. longum* remained stable. Moreover, the number of distinct *Bifidobacterium* species per sample significantly decreased (1.5±0.3 *vs* 2.3±0.3; p = 0.01). Two months after AMC exposure, the mean similarity percentage of TTGE profiles was 55.6% for bacteria and 62.3% for bifidobacteria. These results clearly demonstrated that a common antibiotic treatment may qualitatively alter the colonic microbiota. Such modifications may have potential long-term physiological consequences.

## Introduction

The human colonic microbiota forms a complex ecosystem which plays important roles in human health and disease, with regard to nutrition, pathogenesis and immune function of the host [1]. The composition of the microbiota varies greatly between individuals [2], and for one given individual it can fluctuate as a function of diet, environment [3] and treatments, especially antibiotics [4]. The normal intestinal microbiota provides an important natural defence mechanism against invading pathogens, a process known as barrier effect. Administration of antimicrobial agents causes disturbances in the ecological balance between host and microbes, and between microbes. Mild or severe episodes of antibiotic associated diarrhoea (AAD) are common complications of antibiotic therapy [5]. The major form of intestinal disorders is the pseudomembranous colitis associated with *Clostridium difficile* which occurs in 10–20% of all AAD [6]. Multiple mechanisms occur to protect the individual against enteral pathogens: production of bacteriocins, competitive inhibition for binding to the mucosa, enhancing synthesis of mucus, and secretion of secretory IgA.

Among the colonic microbial populations, the *Bifidobacterium* genus is present in 74% of adult subjects with an average count of 1.6 10^10^ cells/g of feces using culture [7] and near 100% of adult subjects using molecular methods [8]. Bifidobacteria are considered as health-promoting microorganisms due to potential beneficial roles in the intestinal tract, such as immunostimulation, reduction of growth of many potential pathogens and putrefactive bacteria, the prevention of constipation, diarrhoea and other intestinal disorders and improvement of lactose-tolerance, [9], [10], [11]. Moreover, we had previously demonstrated that bifidobacterial increase reduced basal oxidative stress in colonic tissue of healthy rats [12]. *In vitro* susceptibility test showed that the *Bifidobacterium* species isolated from the human colonic microbiota were generally sensitive to amoxicillin [13], [14], [15], [16], a beta-lactam antibiotic widely prescribed for the treatment of respiratory tract infections in adults and children. It has been shown that the fecal microbiota of adults displays a major shift in dominant species upon an amoxicillin treatment, starting 24 h after antibiotic initiation and persisting during treatment [17], [18]. Limited information is available about the effects of oral amoxicillin alone or combined with clavulanic acid on the *Bifidobacterium* species balance [19]. It seems important to assess whether microbial community composition is resistant, resilient or functionally redundant in response to this disturbance. “Resistance” refers to the ability of a community to maintain a given structure in the setting of a perturbation while “resilience” is the ability of a community to return to its baseline structure following a perturbation in community structure [20].

To date, several molecular methods are available to analyse microbial diversity : fingerprinting methods such as Temporal Temperature Gradient gel Electrophoresis (TTGE) and Denaturing Gradient Gel Electrophoresis (DGGE), molecular inventories (PCR, cloning and sequencing of 16S rRNA genes), and more recently, high throughput sequencing such as pyrosequencing. Considering the number of samples to analyse, molecular inventories were not possible. Pyrosequencing could have been used [21],[22]. Nevertheless we decided to use PCR-TTGE since it was less expensive and allowed a greater number of samples to be analysed. Indeed, methods such as TTGE and DGGE based on sequence-specific separation of 16S rRNA gene amplicons, are among the best methods for rapid high throughput comparison of bacterial communities or bifidobacterial species over time [23], [24], [25]. In the present study, we explored, on a 76-day period, the quantitative and qualitative changes occurring in total microbiota and also in the *Bifidobacterium* genus, in 18 adult men after a 5-day amoxicillin-clavulanic acid (AMC) treatment, using specific real-time PCR (qPCR) and PCR-TTGE combined with cloned sequence analysis.

## Materials and Methods

### Bacterial strains and growth conditions

The reference strains used in this study were purchased in lyophilized form from the Pasteur Institute Collection (CIP, Paris, France): *Bifidobacterium adolescentis* CIP64.59^T^, *Bifidobacterium angulatum* CIP104167^T^, *Bifidobacterium bifidum* CIP56.7^T^, *Bifidobacterium breve* CIP64.69^T^, *Bifidobacterium dentium* CIP104176^T^, *Bifidobacterium gallicum* CIP103417^T^, *Bifidobacterium animalis* subsp. *lactis* CIP105265^T^, *Bifidobacterium longum* subsp. *infantis* CIP64.67, *B. longum* subsp. *longum* CIP64.62^T^, *B. longum* CIP64.63 and *Bifidobacterium pseudocatenulatum* CIP104168^T^. Cells were grown for 48 or 72 h, depending on growth rate of the different strains, at 37°C in M20 medium (Pasteur Institute, Paris, France) under anaerobic conditions (Anaerogen™, Oxoid SA, France) before total DNA extraction.

### Clinical data

Eighteen healthy volunteers, non-smoking, 19 to 36 years old men (25±6 years) without any clinical digestive antecedents participated in the study. They did not receive antibiotics or laxatives during the six months prior to the study and gave written informed consent to the protocol which was approved by the local committee on ethical practices ‘Consulting Committee for the Protection of Persons Participating in Biomedical Research’ of Besançon CHU, France.

The 18 healthy volunteers ingested a 875/125 mg oral dose of amoxicillin/clavulanic acid (AMC) twice a day from day 0 to day 4 included. A total of ten fecal samples were collected for each subject over a period of 11 weeks. Firstly, three samples were collected before AMC exposure (day-12, day-6, day 0) to monitor normal fluctuations in fecal bacterial populations. Secondly, fecal samples were taken at the end of AMC exposure (day 5), and during 8 weeks post-exposure (day 8, day 12, day 19, day 26, day 33 and day 64). Samples were collected by the volunteers and stored at 4°C before being transferred to the laboratory where they were stored at −20°C for less than 2 months. Fecal samples were thawed, homogenized and 125 mg aliquot was used to extract total DNA using the chemical guanidium isothiocyanate and the mechanical bead beating method as previously described [24], [26].

### Real-time PCR for total bacteria and *Bifidobacterium* quantification

Reactions were performed in duplicate in a volume of 25 µl within 96-well twin-tech PCR plates, using the Platinum SYBR Green qPCR SuperMix-UDG (Invitrogen, Cergy Pontoise, France). The forward and reverse primers used were Bia339f, Bia788r for total bacteria [23] and Bif164f, Bif662r for *Bifidobacterium* genus [27]. Amplifications were performed in a Mastercycler ep Realplex^4^ (excitation source 470 nm, emission 520/550 nm) (Eppendorf AG, Hamburg, Germany) with the following temperature profile: one cycle at 50°C (2 min) for uracyl-DNA glycosylase action, one cycle at 96°C (2 min), 40 cycles of denaturation at 96°C (15 seconds), primer annealing at 55°C (1 min) for total bacteria and at 62°C (1 min) for bifidobacteria, and elongation step at 68°C (2 min) with fluorescence measure. Finally, the melting curve was made by slowly heating the PCR mixtures from 60 to 96°C (20 min) with simultaneous measurements of the SYBR Green I signal intensities. A standard curve made from known amounts of plasmid DNA containing a 16S rRNA gene insert from *E. coli* or *Bifidobacterium* sp. allowed quantifications.

### Bacteria and Bifidobacteria TTGE

The primers Bia339f and Bia788-GC2r were used to amplify the 16S rRNA genes of total bacteria from samples as already described [28], and the primers Bif164f and Bif662r [27] were used to amplify the 16S rRNA genes of the *Bifidobacterium* genus. PCR amplifications were carried out with a standard PCR mix (0.5 U of *Taq* DNA polymerase (Ampli*Taq* Gold; Perkin-Elmer Corporation, Foster City, Calif.), 1× reaction buffer II, 2.5 mM MgCl_2_, 200 µM of each dNTP and 0.4 µM of each primer in a final volume of 20 µl). Initial denaturation of template DNA and Taq activation were carried out at 94°C for 10 minutes, followed by 35 cycles consisting of 96°C for 15 seconds, 55°C for 1 minute (bacteria) or 62°C for 1 minute (bifidobacteria), 72°C for 4 minutes and a final extension at 72°C for 15 minutes. PCR products were separated on TTGE, using a Dcode™ system (Bio-Rad laboratories, Hercules, CA, USA) and analysed as previously described [23], [24]. The relative front (Rf) was calculated for each band. This parameter is defined as the distance from the top of a defined lane from gel to the band. One or two standards consisting of a mixture of PCR products obtained from identified species or clones were run alongside the fecal samples (M for bacterial TTGE; M1 and M2 for bifidobacterial TTGE). In order to compare samples from different gels and identify the species present in feces, normalization of Rfs for the bands according to the standards was performed. From the bacterial profiles obtained, three bands of interest were excised from different gels, cloned and sequenced as previously described [29]. From the bifidobacterial profiles, eleven bands were excised from gels, cloned and sequenced. The selection criterion for excision was their absence in our Rf data bank or identical Rf in different gels (present or not in our data bank).

Comparisons of TTGE profiles were performed using Dice's similarity coefficient (Dsc) analysis based entirely on the results of band classification [30]. Dsc values were compared, based on the presence or absence of bands. Dice's coefficient is defined as follows: Dsc = [2j/(a+b)] where j is the number of common bands between samples A and B; a and b are the total number of bands in samples A and B, respectively. This coefficient ranges from 0 (no common bands) to 1 (identical bands patterns). TTGE similarities were presented as percentages.

Jaccard's similarity coefficient [30] was also used and is defined as follows: S_AB_ = nS^+^/nS^+^+nd, where nS^+^ is the number of common species between samples A and B; and nd the number of non common species between samples A and B.

### Data analysis

Quantitative data were presented as log_10_ 16S rRNA gene copies/g wet weight of feces. The microbial diversity is assessed through the number of different species per sample ie per microbiota (based on TTGE profiles). All results were expressed as means ± standard error of the mean (SEM). Statistical analyses were performed with Statgraphics plus 5.1 package (Statistical Graphics Corp., Rockville, MD, USA) using the paired T test and the Wilcoxon T test for quantitative data.

## Results

### Quantification of total bacteria and bifidobacteria in fecal samples

**Figure 1:**
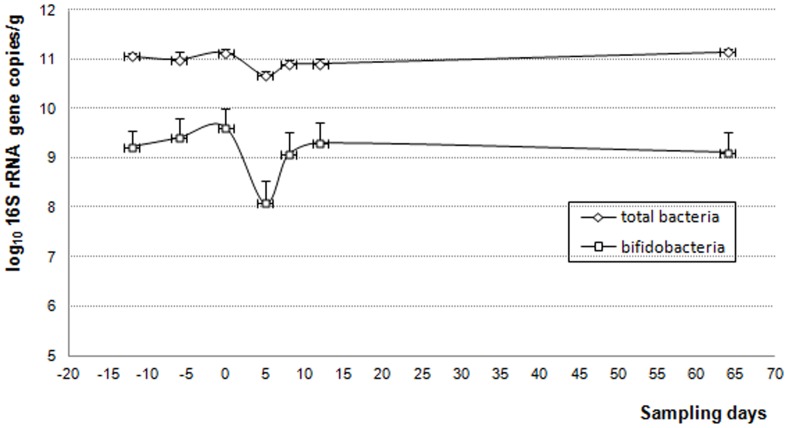
Monitoring of total bacteria and bifidobacterial counts using qPCR before and after AMC exposure (day0–day5).

Mean 16S rRNA gene copy numbers of total bacteria per gram of feces were 11.1±0.1 log_10_ before AMC exposure, 10.7±0.1 log_10_ (p = 0.003) at the end of AMC treatment (day 5) and returned to baseline at day 8 (10.9±0.1 log_10_) (Fig. 1). All fecal samples contained bifidobacterial 16S rRNA gene copies ranging from 4.8 to 11.2 log_10_. Before AMC exposure, only two subjects had bifidobacterial counts ≤6.2 log_10_ copies/g and showed low counts after. Six subjects had marked decrease of bifidobacterial number at day 5 and the 10 other subjects showed no significant quantitative change at the same period. Mean 16S rRNA gene copy numbers of bifidobacteria per gram of feces were 9.4±0.3 log_10_ before AMC exposure and 8.1±0.5 log_10_ (p = 0.003) at day 5. In percentages of total bacteria, there was no significant difference at day 5 (7.4±2.6% *vs* 12.6±2.6% at day 0). Total bifidobacteria 16S rRNA gene copy numbers also returned to baseline at day 8 (9.1±0.5 log_10_ copies/g).

### Impact of antibiotic therapy on dominant fecal microbiota

**Figure 2:**
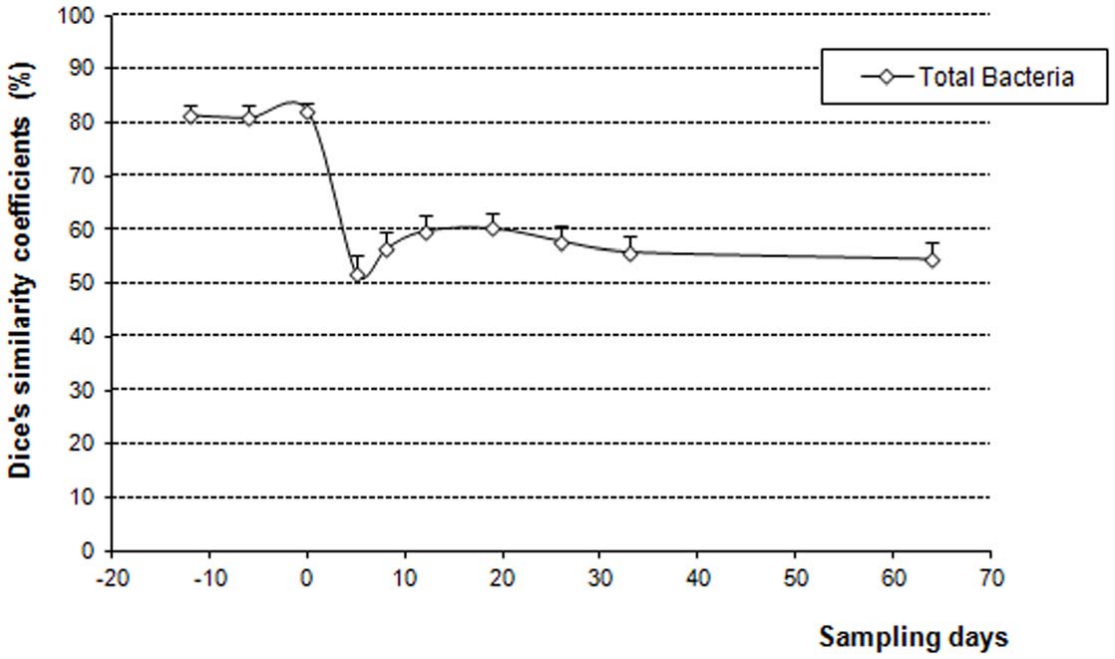
Mean similarity coefficients of TTGE profiles of samples collected before and after AMC exposure (day0–day5) and calculated according to the reference period (day-12 day-6 day 0; n = 18).

TTGE provided a rapid overview of changes in dominant members of the community. The intra-individual similarity indices of the TTGE profiles were calculated, considering day -12, -6 and 0 as reference period. Mean similarity percentage of pre-exposure period was 81.4%±2.1% (Fig. 2). Therefore, before AMC exposure, the bacterial profiles of each subject seemed sufficiently stable to allow observation of changes associated with AMC administration.

**Figure 3:**
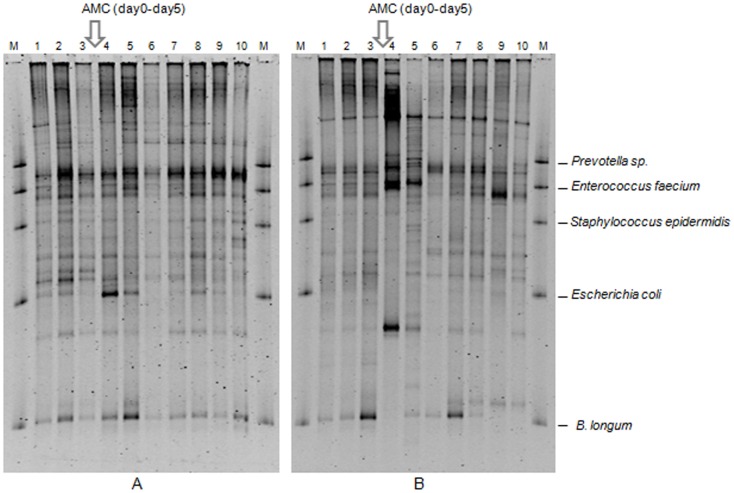
TTGE profiles representing the bacterial diversity of fecal samples from two healthy volunteers (A and B) before and after AMC exposure. 1: day-12; 2: day-6; 3: day 0; 4: day 5; 5: day 8; 6: day 12; 7: day 19; 8: day 26; 9: day 33; 10: day 64.

The TTGE profiles were composed of 2 to 17 bands. The database set which was developed for these samples contained a total of 53 unique bands, which were found in different combinations among the TTGE banding patterns. At day 5, mean similarity percentage decreased significantly to 51.6%±3.5% (p<0.01). One and two months after the end of AMC treatment, the mean similarity percentages of TTGE profiles were 55.9% and 55.6% respectively, showing that total microbiota did not return to its baseline (Fig. 3). Sequencing of three dominant bands leads to the identification of *Ruminococcus* sp (Rf 0.50), *Escherichia coli* (Rf 0.59) and *Bifidobacterium* sp (Rf 0.90). These bands were frequently retrieved and appeared or disappeared according to the antibiotic treatment. The relative intensity of some bands decreased significantly with the AMC treatment as bands comigrating with *Bifidobacterium* sp. Conversely, the relative intensity of bands comigrating with *E. coli* or other Enterobacteria increased significantly at day 5. Bands comigrating with *Ruminococcus* sp. were not affected by the treatment.

### Impact of antibiotic therapy on bifidobacterial microbiota

**Figure 4:**
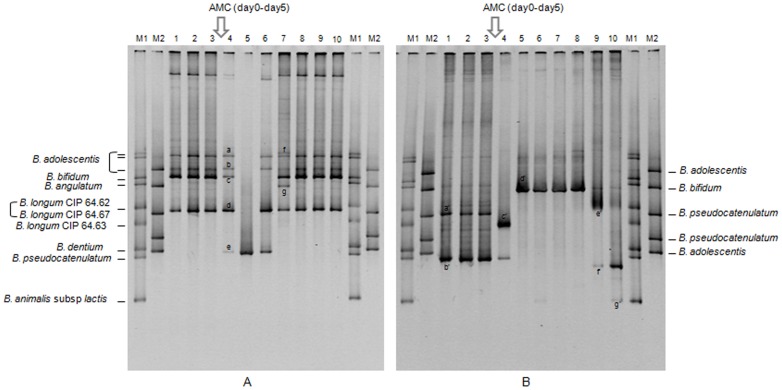
TTGE profiles representing the bifidobacterial diversity of fecal samples from two healthy volunteers (A and B) before and after AMC exposure. 1:day-12; 2:day-6; 3:day 0; 4:day 5; 5:day 8; 6:day 12; 7:day 19; 8:day 26; 9:day 33; 10:day 64. a, b, d, e, f, a′: *B. adolescentis*; c, g: *B. bifidum*; b′: *B. pseudocatenulatum*; c′: *B. longum*; d′: *B. dentium*; e′: *B. breve*; f′: *B. pseudolongum*; g′: *B. animalis* subsp. *lactis*.

**Figure 5:**
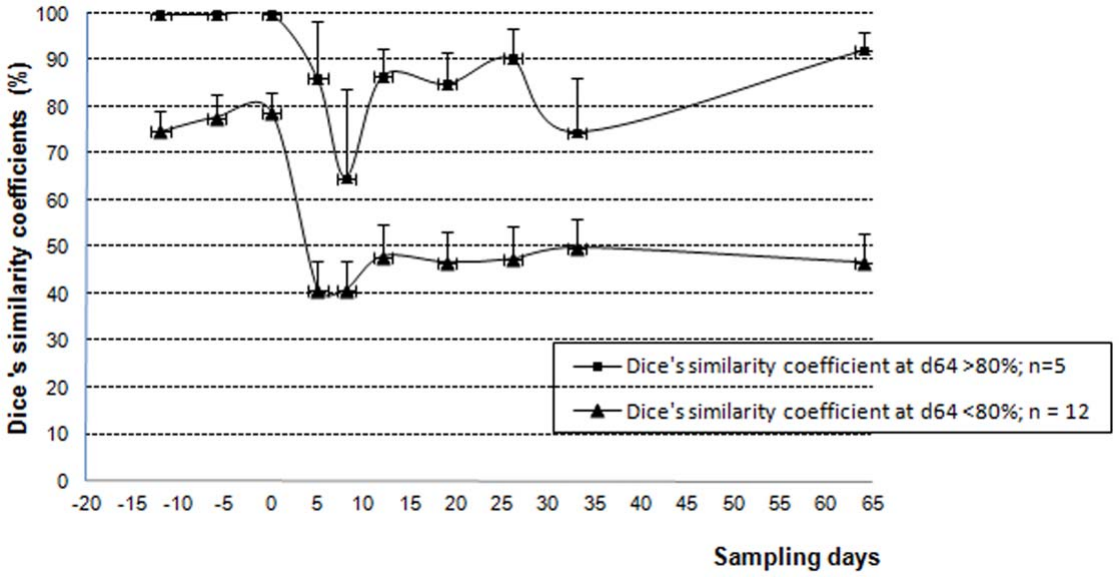
Mean similarity coefficients of specific *Bifidobacterium* TTGE profiles of samples collected before and after AMC exposure (day0–day5) and calculated according to the reference period (day-12 day-6 day 0; n = 17).

The bifidobacterial TTGE profiles were composed of 1 to 7 bands. Only one subject failed to present TTGE profiles due to a weak cell amount (about 4.8 log_10_). Three types of TTGE profile evolutions were observed over time: some microbiota did not change during the whole study (n = 3), a few showed a return to baseline after being changed by antibiotics (n = 5) (Fig. 4A), and for the others, the species balance perturbed at day 5 did not return to baseline at day 33 nor at day 64 (n = 9) (Fig. 4B). The intra-individual similarity indices of the TTGE profiles were calculated, considering day -12, -6 and 0 as reference period. Mean similarity percentage within reference period was 84.5%±4.1%. Only seven subjects presented a 100% similarity between day-12, day-6 and day 0, but twelve subjects presented a 100% similarity between day -6 and day 0 and little variations occurred for the others. At day 5, mean similarity percentages were 55.8%±7.6%. The microbiota of three subjects did not respond to AMC treatment (Dice's similarity coefficient 100% at day 5 and day 8), whereas there was a marked change of TTGE profiles for the others. One or two months after the end of AMC treatment, the mean similarity percentages of TTGE profiles were 59.6% and 62.3% respectively, showing that microbiota was still modified. To improve comparisons, two groups were constituted. The first gathered volunteers with Dice's similarity coefficients at day 64≥80% (corresponding to the mean observed during reference period (n = 5)) and the others (n = 12) (Fig. 5). The microbiota of three individuals within the 5 showed marked alterations of TTGE profiles at day 5 (or day 8) and returned to similarity percentages ≥90% at day 64. The last two microbiota were resistant to AMC. In this group, the microbiota studied in the reference period was very stable (100% similarity). Among the other volunteers microbiota, a Dice similarity coefficient between 0 and 78% was found on day 64 (Fig. 4B). The TTGE profiles from *Bifidobacterium* species of first or second groups were not specific to one group.

To check if this profile variation corresponded only to strain change or to species change, identification of bands were realised. Most fragments co-migrated to the same position as reference strains or clones (M1 and M2) but a few migrated to different positions and could not be identified in this way. Many cultured collection bifidobacteria and many clones are present in our data bank. Recently, twenty six bands were cloned and sequenced [29]. In this study, band sequencing of 11 new bands resulted in the characterization of *B. dentium* (normalised relative front (Rf) 0.50; 0.63; 0.77), *B. pseudolongum* (Rf 0.76) and the confirmation of *B. longum* (Rf 0.62; 0.63), *B. adolescentis* (Rf 0.58; 0.72), *B. pseudocatenulatum/B. catenulatum* (Rf 0.74), *B. lactis* (Rf 0.90). Rf 0.63 corresponded to two possible identifications (*B. dentium* or *B. longum*) but band identified as *B. dentium* was always thinner than *B. longum*.

**Figure 6:**
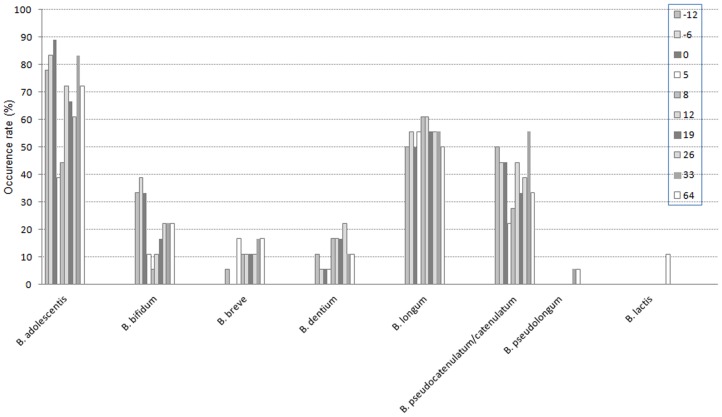
Occurence rate of *Bifidobacterium* species monitored before and after AMC exposure (day0–day5); n = 18 adults.

Similar profiles were observed at day -12, day -6 and day 0 (Fig. 6) with little variation as illustrated by Dice's TTGE coefficients. AMC treatment (day 5) did not affect *B. longum* (TTGE bands found in 56% of subjects versus 52% during reference period) or *B. dentium* (6% in both period) but induced a significant decrease of *B. adolescentis* (39% versus 83%), *B. bifidum* (11% versus 35%) and *B. pseudocatenulatum/B. catenulatum* group (22% with a disappearance in 6 subjects and an appearance in 2 other subjects, versus 46%). One *B. breve* band appeared and was detected in 3 subjects (17%). Moreover, the average number of *Bifidobacterium* species per sample was significantly lower compared to the pre-exposure period (1.5±0.3 *vs* 2.3±0.3) (p<0.05).

At day 8, the average number of *Bifidobacterium* species per sample was 1.7±0.3 and increased up to 2.1±0.3 at day 12 (not significantly different from day 0). At day 8, the species distribution was similar to day 5 for the 18 subjects. At day 12, *B. adolescentis* increased significantly (72%), as *B. pseudocatenulatum/B. catenulatum* (44%). *B. bifidum* did not change (11%). At day 33, TTGE bands corresponding to Rf of *B. adolescentis* were detected in 83% of subjects, Rf of *B. longum* in 55% of subjects, Rf of *B. pseudocatenulatum/B. catenulatum* group in 55% of subjects, Rf of *B. bifidum* in 22% of subjects, Rf of *B. dentium* in 11% of subjects and those of *B. breve* in 17% of subjects. At day 64 (2.2±0.2 species per sample), only few differences were still observed in species identification.

**Figure 7:**
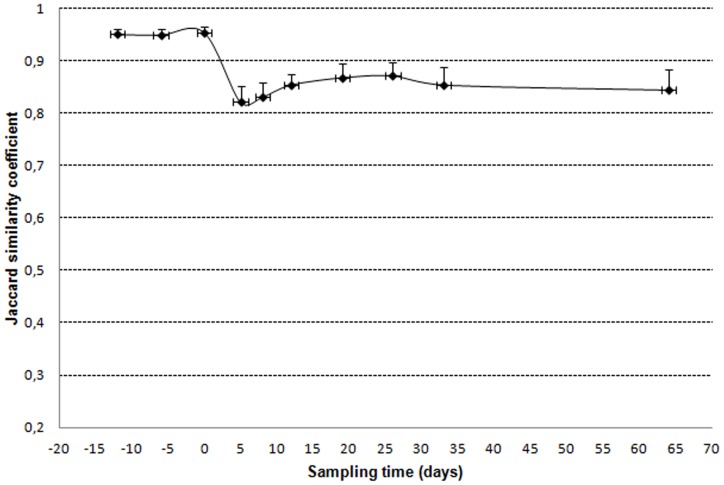
Change of species after the AMC exposure.

Since *Bifidobacterium* bands were all identified, the Jaccard's similarity coefficient equivalent to Dice's was calculated with species as characters and showed that changes concerned species and not only strains within a species (Fig. 7).

## Discussion

A previous study on the same 18 volunteers focusing only on bacterial resistance showed that the administration of AMC increased the amoxicillin resistant *Escherichia coli* and enterococci. From a baseline of less than 1%, more than 30% of resistant *E. coli* and 6% of resistant enterococci were observed and progressively decreased to baseline at day 12 [31].

In the present study, the impact of a 5-day AMC treatment was quantitatively and qualitatively assessed at short- and long-term on total microbiota and on fecal *Bifidobacterium* species in 18 healthy men. Bifidobacteria were detected in all fecal microbiota from the 18 subjects (100%) before exposure to AMC. The counts of total bacteria and total bifidobacteria were significantly altered by AMC at day 5 and returned to baseline at day 8. Numerous publications report modifications of fecal microbiota after AMC treatment but only at genus or phylogenetic group level. An increase of enterobacteria was notably described as well as an increase of *Bacteroides* and a decrease of *Clostridium coccoides* phylogenetic groups compared to the control microbiota [32], [33]. At the species level, using clone library comparison, *Bacteroides distasonis* replaced members of *B. fragilis* group in microbiota of a 39 year-old subject, no sequences corresponding to *Clostridium coccoides* group or to *Bifidobacterium* spp. were detected at day 4 of AMC treatment, whereas these two groups represented a third of the sequences detected on day 0. Conversely, 34% of sequences detected on day 4 were members of *Enterobacteriaceae* which represented only 2% of the day 0 sequences [34]. In this case, bifidobacteria were not recovered at day 24, two weeks after the end of the AMC treatment.

In the present study, bacterial TTGE were performed to monitor bacterial species over time and to determine if intestinal microbiota were resistant and/or resilient. Before AMC exposure, mean similarity between samples was 81.4%±2.1%. Thus the TTGE profiles demonstrated a relatively stable banding pattern before AMC treatment as in a previous study on dog microbiota monitored using DGGE during 10 days before an amoxicillin treatment [35]. Only a few bands showed differences in position and intensities. In another study using DGGE, stability was monitored over 4 or 8 weeks on two patients and demonstrated similar data (similarity indices, 83% for one and 90–94% for the other) [18]. Similarity values ranging from 82.6% to 92.5% were obtained for four other subjects over a 6 week period using bacterial TTGE with the V_3_ hypervariable region of 16S rRNA gene [36]. In the present study, mean similarity percentage of TTGE profiles decreased significantly at day 5 (51.6%, at the end of AMC exposure) in comparison to reference period with an increase of intensity for band corresponding to *E. coli* and a decrease of those corresponding to *Bifidobacterium* and *Enterococcus faecium*. Other band identifications could not be performed since more than one species could be identified from one TTGE band as shown using comigrations. In agreement with our study, similarities of 34–42% and 19–27% in comparison to day 0 were previously obtained using DGGE on two patients during therapy with AMC [18]. In the present study, the average similarity remained low (55%) even two months after the end of the AMC treatment. A previous study using the TTGE technique demonstrated the resilience of the dominant fecal microbiota from 5 out of 6 adults 30 days following amoxicillin treatment with average similarities to day 0 of 88% for 5 subjects and below 70% for the last subject [17]. Conversely, for dogs submitted to 7-day amoxicillin treatment, pre-exposure DGGE samples were clearly separated from post-exposure samples collected 2 weeks after treatment, showing no resilience of the intestinal microbiota after the antibiotherapy [35]. Likewise, the *Bacteroides* community never returned to its original composition 2 years after a 7-day clindamycin treatment whereas in the control group, only minor variations in the number of clones were seen [37]. A partial resilience was obtained for three individuals 30 days after a 5-day ciprofloxacin treatment, with a community composition closely resembling its pretreatment state, but several taxa failed to recover up to 6 months after the antibiotherapy [21]. In a second study, the same authors described an incomplete recovery and individualized responses of the human distal gut microbiota to repeated ciprofloxacin perturbation [38]. Long-term impacts were also seen in the gut microbiota after short-term antibiotic treatment with clarithromycin and metronidazole for up to four years post treatment [22].

Bifidobacterial TTGE were performed to monitor the diversity of *Bifidobacterium* species over time. This specific TTGE was an example of changes occurring among total microbiota. Mean similarity percentage of TTGE profiles during the reference period was 84.5%±4.1%. Seven volunteers out of 17 showed 100% similarity during all the reference period (12 days), indicating a very stable microbiota. Profiles from eight other volunteers have 100% similarity for two hazard samples from reference period and showed slight variations for the last sample. Profiles from twelve volunteers have 100% similarity between day -6 and day 0, just before AMC exposure. Stability of microbiota was not absolute. In another study, monthly monitoring of *Bifidobacterium* species in 6 adults during 8 months, using specific qPCR, showed a very stable bifidobacterial microbiota for 5 volunteers and high variations in the population and composition for the last one [39]. Using bifidobacterial DGGE on samples collected during a 6-week period, Vanhoutte and co-workers showed a close profile grouping for 3 subjects with similarity values ranging from 81.4% to 100%, and one subject with a mean similarity value of 70% [36]. At day 5, in our study, mean similarity percentage of TTGE profiles versus the reference period was 55.8%±7.6%. The microbiota were modified for the most, except for four volunteers, three resistant to AMC and the last perturbed only at day 8. The resistance of some microbiota has been clearly demonstrated. During treatments with cephalosporins, the subjects whose feces exhibited beta-lactamase activity displayed smaller changes in the composition of their fecal microbiota than those observed in subjects without such activity. This was thought to be because the beta-lactamase activity destroyed the antibiotic residues that reach the colon during treatment, thus reducing alterations in the microbiota to a minimum [40], [41]. In spite of clavulanic acid presence which is a beta-lactamase inhibitor, remaining intestinal beta-lactamases from individual microbiota could influence the amount of beta-lactam present in the feces during AMC exposure and explain the resistance to changes of some microbiota.

Similarity percentages of TTGE profiles at day 33 and day 64 were 59.6% and 62.3% respectively, showing that microbiota did not return to baseline.

Cloning and sequencing were performed to identify bands of interest and to evaluate if the changes of bands corresponded to changes of species or of strains within the same species. The same identifications were obtained for bands with identical Rf. In agreement with previous studies [7], [8], [19], [42], *B. adolescentis* (83%), *B. longum* (52%) and the *B. pseudocatenulatum/B. catenulatum* group (46%) were the most frequent predominant bifidobacterial species present in adult microbiota followed by *B. bifidum* (35%).

The mean number of *Bifidobacterium* species per sample harbored in dominant microbiota is significantly lower at day 5 (1.5±0.3) compared to reference period (2.3±0.2) (p<0.05). In another study, the average number of species detected per individual were 2.8±1.2 in healthy adults [8]. Furthermore, at day 5, significant alterations for some *Bifidobacterium* species were observed: for example, occurrence of *B. adolescentis* decreased significantly (39% versus 83% in reference period). In some cases, species not present at day 0 and probably belonging to the subdominant microbiota, became dominant, eg *B. longum* or *B. breve*. The occurrence of *B. longum* remained stable after the antibiotherapy. As enlightened in previous studies, the antimicrobial effect is dose-dependent and amoxicillin showed variable MIC (minimum inhibitory concentration) depending on species or strains tested [13], [16]. Generally, *B. adolescentis*, *B. bifidum* and *B. pseudocatenulatum* seemed to be more susceptible *in vitro* (MIC range ≤0.06–0.5 mg/L) than was *B. longum* (MIC range ≤0.06–2 mg/L) [13], [16]. Thus, our results could be explained by MIC values, as well as intestinal beta-lactamases from individual microbiota. Similar results were previously obtained within microbiota of infants treated with a 7day-amoxicillin treatment, but long-term impact was not monitored [29].

Jaccard's similarity coefficients indicated that differences between TTGE profiles corresponded to species changes and not only to strains changes (Fig. 4). *B. bifidum* was not entirely recovered at day 33 or day 64 (22% versus 35% during reference period). In a previous study, a molecular monitoring of intestinal *Bifidobacterium* strains in four adults using RFLP and ribotyping, showed little variations 30 days and 90 days after an AMC exposure [19]. Strains detected at day 0 could be detected at day 90 or be replaced by another strain from the same species displaying a different pattern. The *B. bifidum* species detected in three of four subjects at day 0, disappeared from two microbiota at day 90 [19]. By changing the intestinal species balance, antibiotic exposure may lead to a homeostatic imbalance through alterations in expression of intestinal epithelial cells tight junction proteins, mucins, antimicrobial peptides, and cytokines [43]. A study has shown that capacity of bifidobacterial species to stimulate immunity is strain specific (T_H_1, T_H_2 cytokines, no effect) [44], [45], [46]. Only some strains of *B. longum* subsp. *longum*/*infantis* can protect against the lethal infection of *E. coli* O157-H7 by preventing Shiga toxin production in the caecum and/or Shiga toxin transfer from the intestinal lumen to the bloodstream [47].

In our study, profiles of four volunteers at day 64 presented similarity coefficients ≥90% in comparison with reference period and those of three other volunteers were ≥80% corresponding to mean values during reference period. Among them, three microbiota were stable and could be considered as resistant to the AMC treatment and four as resilient.

In conclusion, this study showed that a 5-day AMC treatment reduced the mean 16S rRNA gene copy numbers of total bacteria and of *Bifidobacterium* populations. Even if both returned to baseline values at day 8, qualitative methods showed that AMC can have an impact on species composition and decreased the diversity of *Bifidobacterium* populations. Two months post exposure, resilience could not be observed neither for *Bifidobacterium*, nor for total bacteria, in most of the subjects. The physiological impact of such long-term modification remains to be assessed.
